# Origin and Evolution of the 18th Century Hospital Movement

**Published:** 1913-12-13

**Authors:** 


### I.—THE ORIGIN AND EVOLUTION OF THE 18th CENTURY HOSPITAL MOVEMENT.

At the commencement of the eighteenth century the only public institutions in London for the reception and treatment of the sick poor were St. Bartholomew's and St. Thomas's Hospitals. In the provinces private charity was the sole alternative to parish relief.

The foundation of St. Mary of Bethlehem (Bedlam) was reserved for lunatics; and, if we except the two Lock Out-houses, originally intended for lepers, but then used for venereal cases belonging to St. Bartholomew's, this was the only "special" hospital in the Metropolis.

These three great hospitals had been established by Royal Charter upon earlier religious foundations for the relief and sustentation of the poor of the City of London.

By 1700 this accommodation had become totally inadequate to the requirements of the sick, for while St. Bartholomew's in that year contained about 300 beds, the number of in-patients at any one time at St. Thomas's was in 1690, 250; in 1706, 362; and in 1717, 420 (Golding).

In 1675 it was ordered that the out-patients at St. Bartholomew's should not exceed twenty-five medical and twenty-five surgical cases; in 1700, 100 were allowed; in 1715, 150, and so on (Paget); while at St. Thomas's in 1699 their number was limited to sixty in any one month. These limitations, of themselves connoting the inadequacy of the supply to the demand, were dictated by the necessity for economy which had arisen in consequence of the destruction by fire of much of the property of the charities; and to the same cause may be attributed the introduction in 1686, by Royal command, of maintenance charges for soldiers, and in 1696 (at St. Bartholomew's) for venereal cases received into the Lock Out-houses.

These charges prepared the way for the demand in the early years of the eighteenth century for admission fees from all patients received into the chartered hospitals, the imposition of which constituted a serious grievance to the necessitous poor, for whose benefit those charities were founded; and, as will be seen hereafter, provided a reason, additional to the lack of accommodation, for the establishment of the voluntary subscription hospitals.

The Acts of Elizabeth, formulated to take the place of the earlier monastic dispensation of charity, to put an end to begging, and to systematise the relief of the poor, had authorised the provision by each parish of work for the able-bodied and dwelling-houses for the aged and infirm. But all that appears to have been compulsory was the maintenance of the poor, and in the majority of cases little or nothing else was effected. Few, indeed, were the instances in which the able-bodied were well employed; and, instead of houses being erected for those unable to work, their cottage rents were paid by the overseers, they were supported by outdoor relief (2s. 6d. to 3s. 6d. per week), and were attended in sickness by a surgeon or apothecary provided by the parish. Such at first was the ordinary course.

Somewhat later, in consequence of further legislation, numerous poor-houses were erected by private benefaction, some of them arising upon older charitable foundations, while others were leased, purchased, or built by trustees with money contributed by the parishioners. Apart from those with private endowments, all of these were supported by the rates. But, in spite of these, in some places the impotent had been allowed to perish from want, while in others applicants able and willing to work had been supplied with monetary relief in lieu of employment. The Elizabethan poor laws had failed.

During the latter half of the seventeenth century several proposals were published for the better provision of work for the able-bodied, in which the impotent appear to have received little consideration. By the existing law, every parish was charged separately to maintain its own poor; but since few parishes were in the financial position to build, or even lease, and establish workhouses for themselves, little was done except in those cases where a special Act was obtained to enable two or more parishes to be conjoined for that purpose. This method, inaugurated at Bristol in 1696, was adopted by fourteen other towns and cities.

But, unlike our present institutions, these were residentiary working-houses for the able-bodied, the aged and infirm not being received into them at first, though they were the only persons for whom the general Acts specially authorised a habitation. So matters continued until the Act 9 George I. gave a further impetus to the workhouse movement by providing for the union of parishes, and by ordering that any person who refused to enter the house should be ineligible for parish relief.

Upon the erection of a country workhouse a surgeon or apothecary was usually engaged to attend the inmates at a salary averaging from £12 to £20 per annum, which included the supply of medicines. Physicians then were few and far between, and where they were available their fees were beyond the means at the disposal of the majority of overseers, with the result that their services were not generally retained. The condition of surgical practice at the same period may be estimated from the fact that one of the arguments adduced in favour of the erection of county hospitals in 1736 suggested that they would obviate the necessity for the long journey in a waggon to London, which was then the lot of those unfortunate patients who required surgical operation.

In most cases rooms were set apart as an infirmary for the use of the sick, who were nursed by certain of the female inmates, from whom came recruits for the staffs of the hospitals; but cases of smallpox and "pestilential distempers" were generally boarded out of the house at the expense of the authorities. In London the infirmaries were more often separate buildings, in which special wards were provided for foul disease, smallpox, and infectious cases; the popular fear of the spread of infection dictating the selection of sites for these infirmaries in isolated spots, such as burial grounds and pest-fields—a recrudescence of the old doctrine of segregation of the leper—regardless of the welfare of the inmates.

Although originating in the sixteenth century, it was not until the Act of 1723 that there was any organised attempt to increase the number of workhouses, and even then so gradual was the progress of the movement that by 1732 only 115, whether serving single or united parishes (unions), existed in Great Britain, 50 of which were in London, and one only in Scotland (Glasgow). It was the failure to procure adequate legislation for the medical treatment of the sick poor which induced the private effort, commenced by the establishment of dispensaries by the College of Physicians, continued by charitable societies such as that at Westminster, and consummated by the erection of the voluntary subscription hospitals. With these we have now to deal.

